# *Ecklonia cava* Extract Exerts Anti-Inflammatory Effect in Human Gingival Fibroblasts and Chronic Periodontitis Animal Model by Suppression of Pro-Inflammatory Cytokines and Chemokines

**DOI:** 10.3390/foods10071656

**Published:** 2021-07-17

**Authors:** Jae-In Jung, Seonyoung Kim, Seung-Min Baek, Soo-Im Choi, Gun-Hee Kim, Jee-Young Imm

**Affiliations:** 1 Department of Foods and Nutrition, Kookmin University, Seoul 02707, Korea; shinseo23@naver.com (J.-I.J.); clsrn3423@naver.com (S.K.); doriya50@naver.com (S.-M.B.); 2 Plant Resources Research Institute, Duksung Women’s University, Seoul 10326, Korea; langdeveu@naver.com (S.-I.C.); ghkim@duksung.ac.kr (G.-H.K.)

**Keywords:** *Ecklonia cava* extract, human gingival fibroblasts, pro-inflammatory cytokine, chemokine, reactive oxygen species, ligature-induced periodontitis rat

## Abstract

Periodontitis is one of the most common chronic inflammatory diseases. The anti-inflammatory effect of the extract from brown algae *Ecklonia cava* was analyzed in lipopolysaccharide (LPS)-stimulated human gingival fibroblasts (HGF-1), the most abundant cells in gingival tissue. The gene expressions of cyclooxygenase-2 and interleukin-6 were decreased by 78 and 50%, respectively, at 100 μg/mL *Ecklonia cava* extract (ECE) treatment. The gene expressions of matrix metalloproteases (MMP-2 and MMP-8) and chemokines (macrophage inflammatory protein 1-alpha and stromal cell-derived factor 1) were also significantly down-regulated by ECE treatment (*p* < 0.05). The increased reactive oxygen species (ROS) production in HGF-1 cells by LPS stimulation was decreased by 30% at 100 μg/mL ECE treatment. The mitogen-activated protein kinase pathway and the nuclear factor-kappa B (NF-κB) signal activated by ROS were suppressed by ECE in a dose-dependent manner. ECE treatment (400 mg/kg, 8 weeks) significantly improved alveolar bone resorption in the ligature-induced chronic periodontitis rat model. ECE supplementation also lowered elevated mRNA expression of the receptor activator of nuclear factor-kappa B (RANKL)/osteoprotegerin (OPG) in the gingival tissue (*p* < 0.05). Therefore, ECE mitigated gingival tissue destruction and bone resorption associated with chronic periodontitis condition.

## 1. Introduction

Periodontitis is one of the most common chronic inflammatory diseases, causing periodontal tissue damage, tooth agitation, and alveolar bone loss. Periodontitis is caused by the result of interaction between biofilm and host immune response. Dysbiosis in oral cavity increases the virulence expression of pathogenic bacteria such as *Porphyromonas gingivalis* and triggers destructive inflammation [1]. Periodontitis-mediated tissue destruction is initiated by the interaction of bacterial components with immune receptors, such as toll-like receptors (TLR), present in immune cells [2]. TLR2 and TLR4 stimulated by lipopolysaccharide (LPS) induce an immune response through the production of pro-inflammatory cytokines such as interleukin-6 (IL-6) and tumor necrosis factor-alpha (TNF-α) [3,4]. Although the exact molecular mechanisms remain unclear, inflammatory cytokines, arachidonic acid metabolites, chemokines, proteolytic enzymes, and oxidative stress has been suggested as a key players in the pathogenesis of periodontitis [5,6].

Human gingival fibroblasts (HGF) are residential periodontal cells surrounding the alveolar bone and, similar to other fibroblasts, are actively involved in tissue homeostasis and the inflammatory process [7]. HGF is known to secrete inflammatory cytokines and chemical mediators, such as IL-1β, IL-6, and TNF-α under immune-stimulated conditions [8,9]. The increased mRNA expression of inflammatory cytokine (IL-8) and chemokine (CXCL8) in HGF-1 cells were decreased by treatment with anti-inflammatory compounds, such as cyanidin-3-glucoside [10].

Seaweed is not only a valuable food resource and excellent source of bioactive compounds to mitigate various human diseases. The brown algae (Lamninariaceae) *Ecklonia cava* (EC) which occurs widely in coastal areas of Korea, China, and Japan contains a variety of phytochemicals, including fucoidans and phlorotannins [11]. Phlorotannins are oligomeric polymers consisting of phloroglucinol (1,3,5-trihydroxybenzene) monomer units. Eckol, 6,6′-bieckol, dieckol, and phlorofucofuroeckol were isolated as major phlorotannin components in *E. cava* [12]. Phlorotannins have been demonstrated strong antioxidant activity. Phlorotannins isolated from *E. cava* effectively decreased photo-oxidative stress and melanogenesis induced by UV-B exposure, and dieckol resulted in the strongest antioxidant activity among phlorotannin components [13]. The supplementation of dieckol-rich phlorotannis ameliorated liver dysfunction by inhibiting the production of reactive oxygen species (ROS) and malonaldehyde [14].

In our previous study, *Ecklonia cava* extract (ECE) reduced osteoclast-specific gene expression in receptor activator of nuclear factor-κB ligand (RANKL)-stimulated RAW 264.7 macrophages. ECE suppressed differentiation of osteoclasts via protein kinase (MAPK) and nuclear factor-κB (NF-κB) signaling while promoting heme oxygenase-1 expression [15]. We also demonstrated a strong anti-inflammatory effect of ECE in LPS-stimulated RAW 264.7 macrophages and a periodontitis preventive effect in the ligature-induced rat model [16].

As a continuation of our previous studies, the present study was conducted to examine the effect of ECE treatment on ROS and chemokine production and inflammatory cell signaling in human-derived HGF-1 cells. In addition, the effect of ECE on alveolar bone loss was evaluated in the pre-induced periodontitis rat experimental model.

## 2. Materials and Methods

### 2.1. Materials

Dulbecco’s modified Eagle’s medium (DMEM), fetal bovine serum (FBS), and penicillin-streptomycin were purchased from WelGENE, Inc. (Daegu, Korea). The prostaglandin E_2_ (PGE_2_) ELISA kit was purchased from Cayman Chemical Co. (Ann Arbor, MI, USA). Taqman^®^ Universal Master Mix, Taqman^®^ probes (5′-fluorescein-based reporter dye; 3′- quencher dye TAMRA), and high-capacity RNA-to-cDNA kit were purchased from Applied Biosystems (Foster City, CA, USA). Antibodies (p38, extracellular signal-regulated kinase (ERK), NF-κB, β-actin, and TATA-binding protein (TBP) were obtained from Cell Signaling Technology (Danvers, MA, USA). c-Jun N-terminal kinase (JNK) antibodies were obtained from Santa Cruz Biotechnology, Inc. (Santa Cruz, CA, USA). Other reagents were purchased from Sigma-Aldrich Inc. (St. Louis, MO, USA).

### 2.2. Preparatiom of ECE

After removing impurities, the dried EC was extracted with 70% (v/v) aqueous etha-nol for 4~6 h at 70 ± 10 °C. The extract was filtered and concentrated under pressure. The extract was further purified, concentrated, and dried to obtain powdered extracts.

### 2.3. Cell Culture

HGF-1 cells (ATCC, Manassas, VA, USA) were cultured in DMEM containing 10% FBS (v/v) and 1 % penicillin-streptomycin (100 U/mL) at standard condition (37 °C, 5% CO_2_). The culture medium was changed every 2~3 days. Only HGF-1 cells with passage numbers 4 to 10 were used for the cell culture experiment. Cytotoxicity was measured using the MTT assay [15].

### 2.4. Prostaglandin E_2_ (PGE_2_)

HGF-1 cells were seeded in a 96-well plate at a density of 1×10^4^ cells/well and incubated overnight. Various concentration of ECE (25, 50, and 100 μg/mL) and LPS (5 μg/mL) were added to the cells and incubated for another 24 h. The concentration of PGE_2_ released from the cells in the culture supernatant was analyzed using Cayman ELISA kits.

### 2.5. ROS Analysis

The effect of ECE on ROS production was determined using the fluorescence probe, 2′,7′-dichlorodihydrofluorescein-diacetate (DCFH-DA), as previously described [15]. Briefly, HGF-1 cells were incubated in the presence of ECE for 24 h prior to DCFH-DA (25 μM, 1 h) treatment. After that, the HGF-1 cells were stimulated by LPS for 2 h. The intensity of 2′,7′-dichlorofluorescein was measured using a microplate reader (Biotek Instruments Inc., Winooski, UT, USA) at excitation/emission wavelengths 485/528 nm.

### 2.6. Western Blotting Analyses

The HGF-1 cells were lysed with RIPA lysis buffer (ATTO, Japan) containing phosphatase and protease inhibitor. The cytoplasmic and nuclear proteins were obtained using NE-PER^®^ Nuclear Extraction Reagents (Thermo Scientific, Rockford, IL, USA). The Bradford assay was used for protein quantification. The lysed proteins were separated by 10% SDS-PAGE and transferred onto polyvinylidene fluoride membranes for 1.5 h (Millipore, Billerica, MA, USA). They were treated with primary antibodies (p38, ERK, JNK, NF-κB, β-actin, and TBP) and horseradish peroxidase-conjugated secondary antibodies. Visualization and quantification of target proteins band intensities were done as previously described [17].

### 2.7. Animals

Male Sprague-Dawley rats (6 weeks old, 400~430 g; Orient Bio, Gapyeong, Korea) were accommodated in a cage at 23 ± 3 °C and a 55 ± 15% relative humidity under a 12-h light-dark cycle. All animals had free access to purified water and a standard diet (Cargill, Inc., Seongnam, Korea). Animal experiments were approved and conducted under the guidance of the Institutional Animal Care and Use Committee (KNOTUS 19-KE-398).

### 2.8. Induction of Ligature-Induced Periodontitis

After one week adaptation, the rats were randomly divided into six groups of 6 rats: (1) no ligation, (2) ligation control, (3) ligation positive control (doxycycline 20 mg/kg/day), (4) ligation commercial functional agent control (Insadol 200 mg/kg/day), (5) ligation + ECE low dose (100 mg/kg/day), (6) ligation + ECE high dose (400 mg/kg/day). The experimental dosage was decided based on a report of ECE on postoperative and neuropathic pain relief in rats [18]. The experimental periodontitis was induced for 8 weeks by ligation [16], and sterile distilled water (ligation control), doxycycline, Insadol, and ECE (low and high dose) were orally administered for another 8 weeks, respectively. 

### 2.9. Micro-Computerized Tomography (Micro-CT)

Morphology around the molar tooth and the alveolar bone of animals was observed using the Viva CT 80 scanner (SCANCO Medical, Wangen-Bruttisellen, Switzerland) before (8 weeks) and after ECE administration (16 weeks). The distance between cementoenamel junction (CEJ) and the alveolar bone crest (ABC) was measured as an index of alveolar bone loss.

### 2.10. Quantitative Real-Time PCR (qRT-PCR)

HGF-1 cell lysates and the rat gingival tissues were used as specimens for qRT-PCR. Target gene expressions (COX-2, IL-6, MMP-2, MMP-8, macrophage inflammatory protein 1-alpha (MIP-1α), stromal cell-derived factor 1 (SDF-1), RANKL, and osteoprotegerin (OPG) were analyzed by a StepOne Plus RT-PCR system (Applied Biosystems). The relative changes in target gene expression level were normalized against the house-keeping gene, β-actin.

### 2.11. Statistical Analysis

All cell culture experiment and analytical measurements were conducted in triplicate. Data were presented as mean ± standard deviation (SD). SPSS 25 (SPSS, Inc., Chicago, IL, USA) was used for statistical analysis. One-way analysis of variance with Duncan’s multiple comparison test was carried out to find out differences among treatment means (*p* < 0.05).

## 3. Results and Discussion

### 3.1. ECE Decreases PGE_2_ Production and Pro-inflammatory Enzyme Expression in LPS-Stimulated HGF-1 Cells

**Figure 1:**
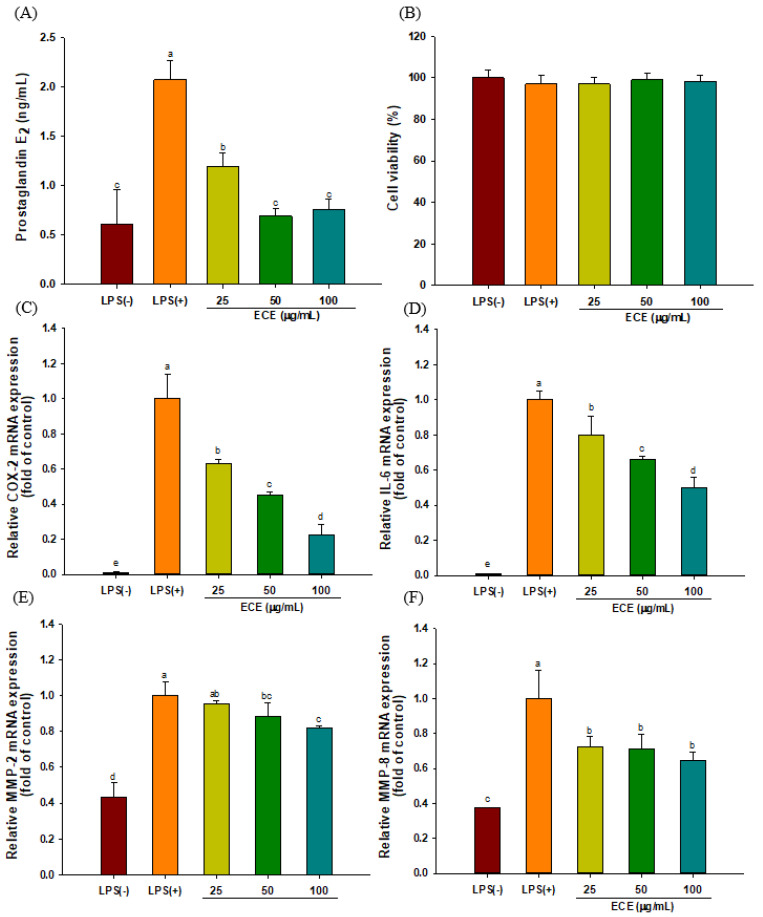
Effect of ECE treatment on (**A**) PGE_2_, (**B**) cell viability, (**C**) COX-2, (**D**) IL-6, (**E**) MMP-2, and (**F**) MMP-8 mRNA expressions in LPS-stimulated HGF-1 cells. Cells were incubated for 24 h in the presence of LPS (5 μg/mL) and ECE. Different letters (a–e) show significant difference (*p* < 0.05). ECE: *Ecklonia cava* extract; PGE_2_: prostaglandin E_2_; COX-2: cyclooxygenase-2; IL-6: interleukin-6; MMP-2: matrix metalloproteinases-2; MMP-8: matrix metalloproteinases-8; LPS: lipopolysaccharide; HGF: human gingival fibroblasts.

The effect of ECE treatment on PGE_2_ production was examined in LPS-stimulated HGF-1 cells. PGE_2_ production was significantly down-regulated by ECE treatment (Figure 1A). No cytotoxic effect of ECE treatment was observed in the tested concentration range (Figure 1B). The level of COX-2 gene expression was significantly increased upon LPS stimulation, while ECE treatment inhibited COX-2 expression in a dose-dependent manner (Figure 1C). The gene expression of IL-6, one of the major players in periodontal inflammation, was down-regulated by 49% following ECE treatment at 100 μg/mL (Figure 1D). The expression levels of MMP-2 and MMP-8 were significantly suppressed by ECE treatment at 50 and 100 μg/mL (Figure 1E,F).

LPS, an outer cell membrane component of Gram-negative bacteria, is a major virulence factor eliciting a host immune response [19]. HGF is a dominant cellular constituent of gingival tissue and secretes inflammatory cytokines, such as IL-6 and PGE_2,_ similar to monocytes and macrophages upon LPS stimulation [8,20]. It has been reported that PGE_2_ production in gingival tissue increases up to 10-fold in periodontal disease [21]. In addition, endothelial dysfunction was induced by elevated COX-2 expression in rats with periodontitis [22]. COX-2 catalyzes the production of PGE_2_ from arachidonic acid, while selective COX-2 inhibitors are effective to retard the progression of periodontal diseases [23].

In our previous study, ECE treatment effectively suppressed inducible nitric oxide synthase (iNOS), TNF-α, and IL-1β mRNA expression in LPS-stimulated RAW 264.7 macrophages [16]. Ha et al. [24] reported that ECE and dieckol purified from ECE effectively reduced airborne particulate-induced PGE_2_ generation in human keratinocytes by inhibiting COX-2, and microsomal PGE_2_ synthase-1 and -2. IL-6 is produced by a variety of cells, such as macrophages, endothelial cells, and fibroblasts, and is closely related to periodontal inflammation [25]. An elevated IL-6 level is associated with various chronic inflammatory diseases, while suppression of IL-6 is effective in mitigating rheumatoid arthritis [26]. Phloroglucinol derived from *E. cava* reduced chronic inflammation by both inhibiting the production of inflammatory mediators (IL-1β, IL-6, TNF-α, and PGE_2_) and oxidative stress in LPS-stimulated HT1080 human fibrosarcoma cells [27].

MMPs are proteolytic enzymes that modulate tissue homeostasis. Although increased MMP activity during the inflammatory state degrades extracellular matrix, MMPs also regulate the progression of inflammation by modulating activity of cytokines and chemokines [28]. MMP-mediated degradation of extracellular protein weakens periodontal ligament and possibly causes tooth mobility and alveolar bone loss. Several collagen-degrading MMPs (MMP-2, -7, -8, and -13) were expressed in gingival epithelium and fibroblasts in periodontitis patients [29]. Thus, it was suggested that broad target MMPs are desirable to reduce periodontal tissue destruction.

Based on our previous report, dieckol-containing ECE also significantly reduced RANKL-induced osteoclastogenesis by downregulating osteoclast-specific transcriptional factors such as NFATc1 and c-fos [15]. The results of current study and our previous reports [15,16] indicate that ECE possesses anti-inflammatory activity that mitigates periodontitis.

### 3.2. ECE Lowers Pro-Inflammatory Chemokine Gene Expressions in LPS-stimulated HGF-1 Cells

**Figure 2:**
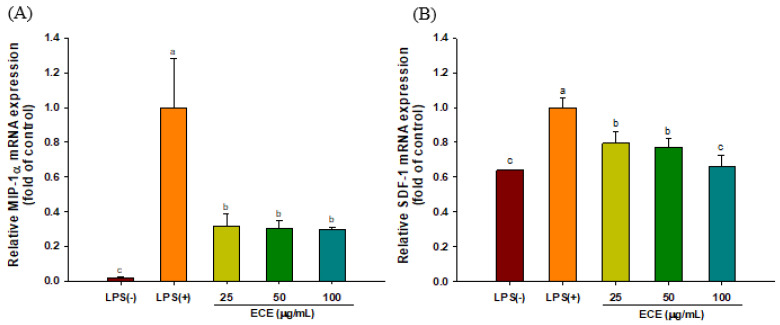
Effect of ECE on LPS-stimulated inflammatory chemokines (**A**) MIP-1α and (**B**) SDF-1 mRNA expressions in HGF-1 cells. Different letters (a–c) show significant difference (*p* < 0.05). ECE: *Ecklonia cava* extract; MIP-1α: macrophage inflammatory protein 1-alpha, SDF-1: stromal cell-derived factor 1. LPS: lipopolysaccharide; HGF: human gingival fibroblasts.

Chemokine is a type of cytokine that recruits neutrophils, macrophages, and lymphocytes. The abundance of chemokines is closely associated with inflammatory periodontal tissue damages [30]. The changes in gene expression of MIP-1α and SDF-1 in response to ECE treatment were analyzed using qRT-PCR. As shown in Figure 2, the gene expression of MIP-1α and SDF-1 was decreased by 63% and 24% with 25 μg/mL ECE treatment, respectively.

MIP-1α (CCL3) is one of the important chemokines that expressed in oral inflammatory disease and it is produced by fibroblast, endothelial cells and osteoblasts [31]. MIP-1α induced the differentiation of osteoclasts by enhancing RANKL expression in bone marrow stromal cells and induced the differentiation of osteoblasts via MAPK and PI3K/Akt pathways [32]. MIP-1α is also involved in neuro-inflammation. The expression of MIP-1α was increased in LPS-injected rats and enhanced the production of COX-2 and accumulation of microglia. The induction of MIP-1α was partially modulated through the MAPK and NF-κB pathway [33].

Although SDF-1 (CXCL12) has been considered as a homeostatic chemokine, it mainly acts as a regulator of leukocyte recirculation and CD4+ T cells activation [34]. Upregulation of SDF-1 and its receptor CXCR4 was noticed in inflamed dental pulp [35]. SDF-1/CXCR4 signal transduction plays an important role in cancers and immune diseases, leading to the suggestion that regulation of the SDF-1/CXCR4 signal could be a potential therapeutic target for the modulation of inflammatory disorders [36]. The stimulation of SDF-1/CXCR4 induced the secretion of MMP-2 and MMP-9 in neural stem cells isolated from embryonic Wistar rats [37]. Rheumatoid arthritis patients showed increased levels of SDF-1, which stimulated the expression of MMP-9 and osteoclast-mediated bone-resorption [38].

### 3.3. ECE Suppresses ROS Production and MAPK Signaling in LPS-Stimulated HGF-1 Cells

**Figure 3:**
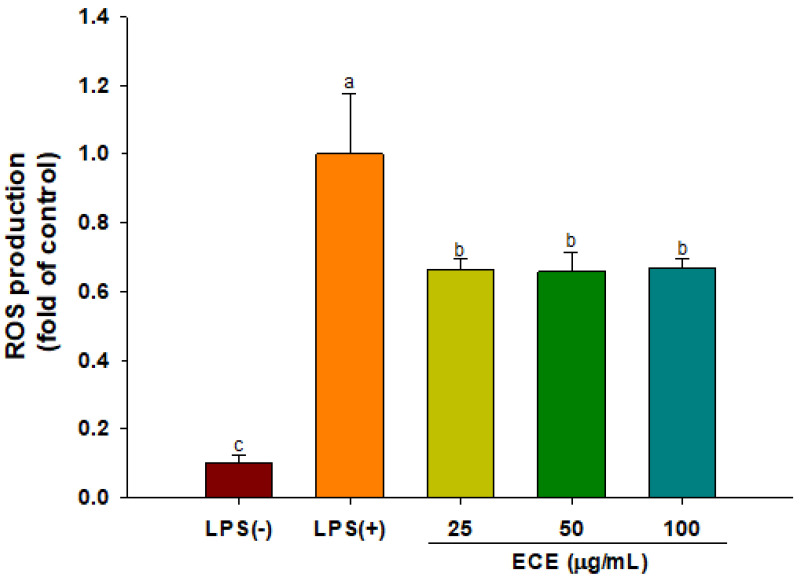
Effect of ECE on ROS production in LPS-stimulated HGF-1 cells. Different letters (a–c) show significant difference (*p* < 0.05). ROS: reactive oxygen species; LPS: lipopolysaccharide; HGF: human gingival fibroblasts.

The changes in ROS production of HGF-1 cells in response to ECE treatment were analyzed using fluorogenic probe, DCFH-DA. LPS stimulation increased ROS production while ECE treatment inhibited ROS production by 30% in HGF-1 cells (Figure 3). The over-production of intracellular ROS might cause mitochondrial dysfunction and eventually leads to gingival tissue damage [39]. ECE relieved UVB-mediated oxidative stress in HaCaT cells and zebrafish. Among phlorotannin constituents, dieckol is considered a major active compound for suppressing UV-induced cell death [40].

**Figure 4:**
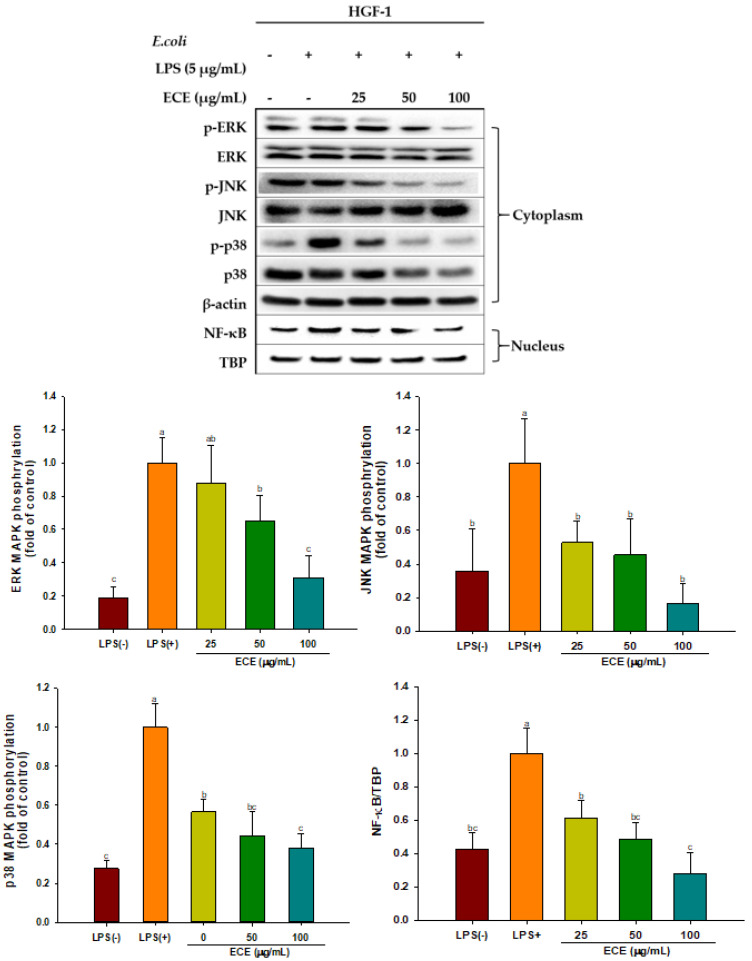
Effect of ECE on protein expression associated with MAPK and nuclear NF-κB in LPS-stimulated HGF-1 cells. Different letters (a–c) show significant difference (*p* < 0.05). ECE: *Ecklonia cava* extract; LPS: lipopolysaccharide; HGF: human gingival fibroblasts. ERK: extracellular signal-regulated kinase; JNK: c-Jun N-terminal kinase; MAPK: mitogen-activated protein kinase; NF-κB: nuclear factor-kappa B; TBP: TATA-binding protein.

ROS activates the MAPK signaling cascade, which is closely related to inflammation, periodontal pathogenesis, and rheumatoid arthritis [41,42]. ECE treatment significantly decreased phosphorylation of MAPK, including ERK, JNK, and p38 kinase, in a dose-dependent manner (Figure 4). MAPK plays a pivotal role in LPS-induced inflammation via the production of inflammatory cytokines. Dieckol showed potent anti-inflammatory activity by inhibiting all three MAPK in amyloid-beta peptide- stimulated PC 12 cells. However, other phlorotannins, such as 8,8-bieckol and eckol only partially suppressed MAPK [43]. Activation of the MAPK pathway stimulated MMP-2 and MMP-9 expression but blocking of ERK and JNK signaling via their inhibitors (PD98059 and SP600125) effectively lowered elevated MMP expressions in kidney proximal epithelial cells [44].

Down-regulation of the MAPK pathway by ECE treatment in the present study suggests that ECE has great potential to reduce an inappropriate inflammatory response and gingival tissue damage.

It is known that MAPK signaling activates nuclear translocation of NF-κB, amplifying the expression of inflammatory cytokines, chemokines, and inducible inflammatory enzymes, such as COX-2 and iNOS [45]. The ECE-mediated decreased NF-κB observed in Figure 4 is probably responsible for the suppression of COX-2, and IL-6 (Figure 2). Oxidative stress is a major contributor in the pathogenesis of chronic diseases. The Nrf-2/ARE pathway is a well-known hormetic pathway and increase tolerance to oxidative stress-mediated diseases upon activation. On the contrary, susceptibility to diseases increases at low or without Nrf-2/ARE activation [46,47]. Phlorotannins in ECE can act as hormetic phytochemicals. They stimulated cellular stress pathway and promoted antioxidant enzyme expression. ECE treatment resulted in Nrf-2 activation and induced HO-1 via upregulation of MAPK pathway [15]. Similarly, inhibition of p38 MAPK/NF-kB signaling followed by iNOS and COX-2 expression in BV2 microglial cells was suggested as a major underlying molecular mechanism for dieckol-mediated neuroprotection [48]. It has been demonstrated that low dose dietary phytochemicals, such as, oleuropein aglycone, effectively improved Parkinson’s disease-related aging symptoms and healthspan in *C. elegans* through hormesis mechanism [49]. After absorption, dietary polyphenols and their metabolites produce low concentration of H_2_O_2_ or ROS affecting cell signaling by interaction with endothelial cell membranes. Hormetic actions exerted by dietary polyphenols vary depending on H_2_O_2_ concentration in the blood (0.25~5 μM) [50].

### 3.4. ECE Improves Alveolar Bone Loss in Ligatured-induced Periodontitis in Rats

**Figure 5:**
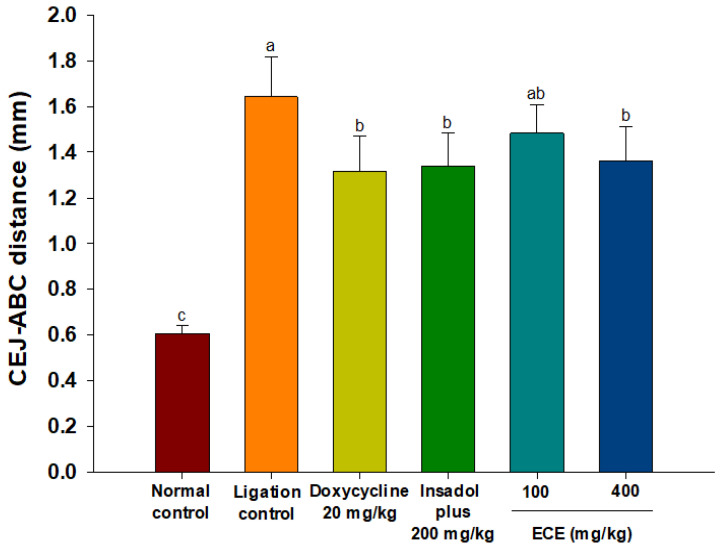
Effects of ECE on CEJ-ABC distance in ligature-induced periodontitis rats. Bars with different letters indicate significant differences (n = 6, *p* < 0.05). ECE: *Ecklonia cava* extract; CEJ-ABC: cement enamel junction-alveolar bone crest.

Alveolar bone loss is a major problem in the progression of periodontitis. To evaluate the efficacy of ECE on bone resorption, periodontitis was induced in rats by ligation for 8 weeks. Periodontal bone height was calculated by the distance from CEJ to ABC by micro-CT analysis. The CEJ-ABC distance of the non-ligation control group was 0.6 mm, while the CEJ-ABC distance reflecting bone loss increased to 1.6 mm in the ligation control group (Figure 5). A significant reduction in the CEJ-ABC distance occurred following administration of the positive control (doxycycline and Insadol) or 400 mg/kg ECE treatment (*p* < 0.05). There was no significant difference between the positive control group and high-dosage ECE group.

Ligature surrounding molar teeth causes plaque accumulation and, eventually, leads to periodontal gum damage and subsequent bone resorption similar to human periodontitis [51]. The experimental setting used in the present study (pre-induction of periodontitis for 8 weeks before sample administration) simulated characteristics of chronic inflammatory disease. The bone resorption observed in the ligature periodontitis model was closely associated with the accumulation of periodontal bacteria in the ligature region [52]. This result indicates that ECE provides a beneficial effect against bone resorption in periodontitis and possibly alleviates disease severity.

Periodontitis is a chronic inflammatory disease and normal vascular function is gradually impaired with continuous inflammation. There is a causal link between endothelial dysfunction and periodontitis [53]. Campi et al. [22] reported notable endothelial dysfunction in ligature-induced periodontitis rats. The administration of dieckol effectively improved endothelial dysfunction in spontaneously hypertensive rats by modulation of the Th17/Treg balance [54].

### 3.5. ECE Lowers Elevated RANKL/OPG Gene Expressions in Gingival Tissues

**Figure 6:**
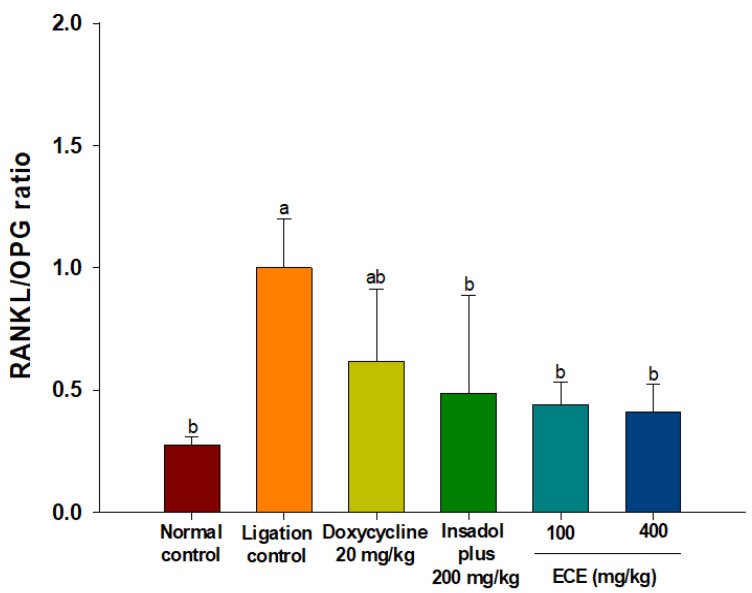
Effect of ECE on RANKL/OPG expression ratio in periodontitis rats. Bars with different letters indicate significant differences (n = 6, *p* < 0.05). ECE: *Ecklonia cava* extract; RANKL: receptor activator of nuclear factor kappa-B ligand; OPG: osteoprotegerin.

The mRNA expression level of RANKL/OPG is considered as a reliable biomarker to manage periodontal disease since RANKL/OPG ratio increases with occurrence of periodontitis [55]. As shown in Figure 6, the RANKL/OPG ratio was significantly increased in all ligature groups compared to the non-ligature control. The administration of ECE, irrespective of the dosage, significantly lowered the RANKL/OPG ratio compared to the ligation control group (*p* < 0.05). The effect of ECE was comparable to that of the positive control.

RANKL stimulates osteoclastogenesis via binding to RANK, the receptor located in the membrane of osteoclast precursor cells. By contrast, OPG, which has structural similarity to RANK prevents interaction between RANKL and RANK by acting as a decoy receptor for RANKL [56]. Consistent with the result of the present study the RANKL/OPG expression ratio was increased in periodontitis patients (n = 15) compared with that of healthy subjects (n = 15) [57]. They found that the expression of RANKL linked with increased number of *Porphyromonas gingivalis* and *Actinobacillus actinomycetemcomitans*. In addition, the elevated RANKL/OPG ratio was strongly correlated with the polarization of T-helper cells to Th17 in gingival tissues [58]. The stimulation of Th17 differentiation promoted NF-κB and exaggerated inflammation with increased alveolar bone loss [59]. This result suggests that ECE-mediated decreased alveolar bone loss is associated with the expression of RANKL/OPG.

## 4. Conclusions

ECE treatment decreased PGE_2_ production and down-regulated the mRNA expressions of pro-inflammatory cytokine (IL-6) and enzymes (COX-2, MMP-2, and MMP-8) in LPS-stimulated HGF-1 cells. Chemokine (MIP-1α and SDF-1) gene expression, which leads to immune cells infiltration to inflamed sites, was significantly down-regulated. The suppression of ROS production and MAPK/NF-κB signaling were contributed to the ECE-mediated anti-inflammatory effect in HGF-1 cells. Administration of ECE significantly improved alveolar bone loss and lowered elevated RANKL/OPG expression in periodontitis gingival tissue. Therefore, ECE has great potential to alleviate gingival tissue destruction and bone resorption associated with the chronic periodontitis condition.
